# A high-resolution beach imagery dataset with COCO annotations for deep learning-based *Sargassum* monitoring in coastal environments

**DOI:** 10.1016/j.dib.2026.112910

**Published:** 2026-05-31

**Authors:** Javier Arellano-Verdejo, Hugo E. Lazcano-Hernandez, Arturo Trejo-Morales, Martin Santos Romero

**Affiliations:** a Department of Observation and Study of the Earth, Atmosphere, and Ocean, El Colegio de la Frontera Sur, Chetumal, Quintana Roo 77014, Mexico; b Department of Observation and Study of the Earth, Atmosphere, and Ocean, SECIHTI-ECOSUR, Chetumal, Quintana Roo 77014, Mexico; c Department of Networks and Information Technology, Universidad Autonoma del Estado de Quintana Roo, Chetumal, Quintana Roo 77019, Mexico

**Keywords:** Pelagic sargasso, Beach imagery classification, Citizen science, Crowdsourcing, Semantic segmentation, Beach monitoring, Mexican Caribbean

## Abstract

Accurate monitoring of *Sargassum* blooms is essential for mitigating their severe socioeconomic and ecological impacts on coastal areas. To support the development of computer vision tools for coastal management, this study presents a dataset of high-resolution images along the coast of Quintana Roo, Mexico. The dataset comprises RGB images, accompanied by pixel-level multi-class segmentation masks. These masks classify the coastal environment into three distinct classes: *Sargassum*, sand, and other environmental elements and coastal objects (such as water, vegetation, sky, clouds, boats, and people, among others). To facilitate immediate integration with modern deep learning architectures, the pixel-level masks have been systematically converted into polygonal annotations formatted as COCO-compatible JSON files. The dataset also includes descriptive statistics detailing class distribution to characterize spatial complexity. This curated, multi-format dataset provides a standardized reference baseline for researchers and developers with which to train, validate, and compare semantic and instance-level segmentation models (e.g., U-Net, Mask R-CNN) aimed at automated quantification of *Sargassum* biomass and efficient coastal cleanup logistics. This dataset goes beyond the previously published collection, making it the current benchmark of its kind.

Specifications Table

**Table:** 

Subject	Earth & Environmental Sciences
Specific subject area	Machine learning for coastal environmental monitoring, Computer Vision, and Pattern Recognition.
Type of data	Raw RGB imagery (.jpg), corresponding mask imagery (.png), and COCO imagery with annotations.
Data collection	The raw imagery was acquired through crowdsourcing [1]. Image segmentations were performed manually to identify Sargassum on the beach [2]. For COCO annotations, a Python script based on OpenCV was implemented to convert segmentation masks into polygonal annotations; the simplified geometries were structured in JSON format according to the Microsoft COCO standard [3].
Data source location	The dataset was built through a crowdsourcing platform called Collective View. The dataset comprises photographs taken along the beaches located in the Yucatan Peninsula, mainly in the State of Quintana Roo, Mexico.
Data accessibility	Repository name: figshare Data identification number: 32125996 Direct URL to data: https://doi.org/10.6084/m9.figshare.32125996 Instructions for accessing these data: Select the direct URL; no figshare account or any other credentials are required to download the dataset.
Related research article	This article uses the dataset used in [2], to construct a new dataset in the Microsoft COCO standard.

## 1 Value of the Data

• This data provides the first high-resolution, open-source, annotated reference dataset specifically designed for the automated monitoring and quantification of *Sargassum* washed up on beaches, contributing to the current state of-the-art in remote sensing of *Sargassum* along coastlines.
• The dataset mathematically captures the class imbalance and fragmentation of real-world coastal environments, making it a highly valuable benchmark for testing advanced loss functions tailored to small-object detection.
• Computer vision researchers and data scientists can use this data to directly train, validate, and compare state of the art semantic and instance segmentation architectures (e.g., U-Net, Mask R-CNN) without having to resort to the costly process of manual image labelling. Baseline performance models utilizing this data layout can be found in [2].
• Government and civil society institutions can leverage models trained on this data to develop accurate early warning systems, automate biomass volume estimation protocols, and design data-driven mitigation strategies for coastlines severely affected by macroalgal strandings.

## 2 Background

*Sargassum* (*S. fluitans* morphotype III and *S. natans* morphotypes I and VIII) is a macroalga that floats freely on the sea surface. Its massive arrival along the beaches of various countries with coastlines on the Caribbean Sea has become a recurring phenomenon since 2018 and currently poses a threat to the ecosystems and coastal communities of that region [1,2]. Monitoring *Sargassum* on beaches is a task that requires periodic information at scales of approximately one meter, making it difficult to implement traditional remote sensing methodologies (satellite or aerial imagery). Consequently, datasets of images showing *Sargassum* on the beach are scarce. To contribute to the monitoring of beaches affected by *Sargassum* blooms, based on a crowdsourcing scheme, a dataset of in situ images of the beach with *Sargassum* was constructed [2]. Subsequently, 1000 images were selected and manually segmented [2]. Finally, based on the segmented dataset, to facilitate the integration of the dataset with modern deep learning architectures, this study provides COCO-compatible JSON files from the *Sargassum* on the beach dataset.

## 3 Data Description

**Fig. 1:**
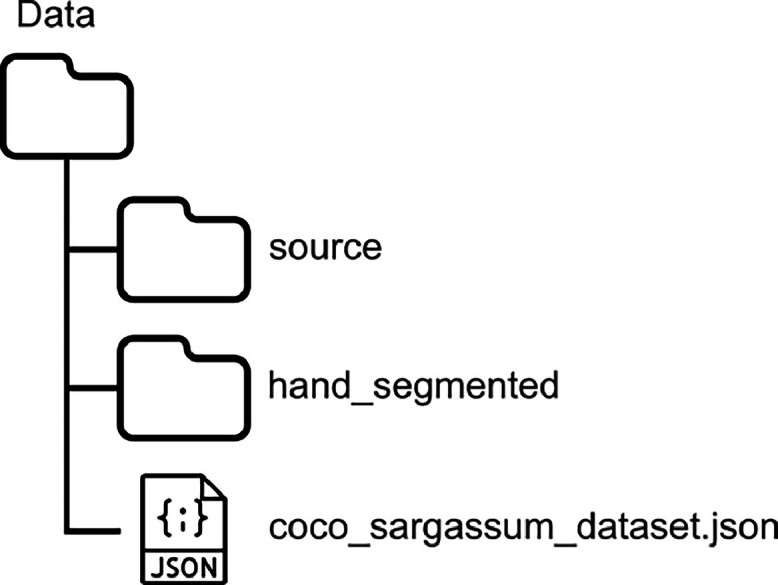
Overview of the dataset folder’s structure.

The dataset presents a curated collection of 1000 in situ images of beaches with *Sargassum*, captured along the Yucatán Peninsula, Mexico, using the citizen science crowdsourcing platform Collective View [1]. The total size of the dataset is 282.60 MB, structured into three main components for each record: original raw images in RGB format (.jpg), manually created semantic segmentation masks (.png), and a unified polygonal annotation file in JSON format. Fig. 1 depicts an overview of the dataset folder’s structure.

For the classification of the coastal environment, the data is divided at the pixel level into three specific semantic categories. The Table 1 describes the exact correspondence between the defined environmental categories (*Sargassum*, Sand, and Other), their RGB pixel values within the semantic segmentation masks, and the integer category identifiers assigned in the COCO individual polygonal instances, commonly known as annotations.

**Table 1:** Class assignment dictionary and technical specifications.

Class	Color	RGB value	COCO Category ID
*Sargassum*	Dark red	[139, 0, 0]	1
Sand	Yellow	[255, 255, 0]	2
Other	Grey	[192, 192, 192]	3

At the instance level, the dataset captures the spatial fragmentation and class imbalance features of real-world coastal environments. The “*Sargassum*” class exhibits the greatest morphological complexity, accounting for a total of 12,308 independent polygonal instances throughout the dataset, followed by the ‘Sand’ class and, finally, the “Other” class, which tends to group the largest contiguous seabed regions.

**Fig. 2:**
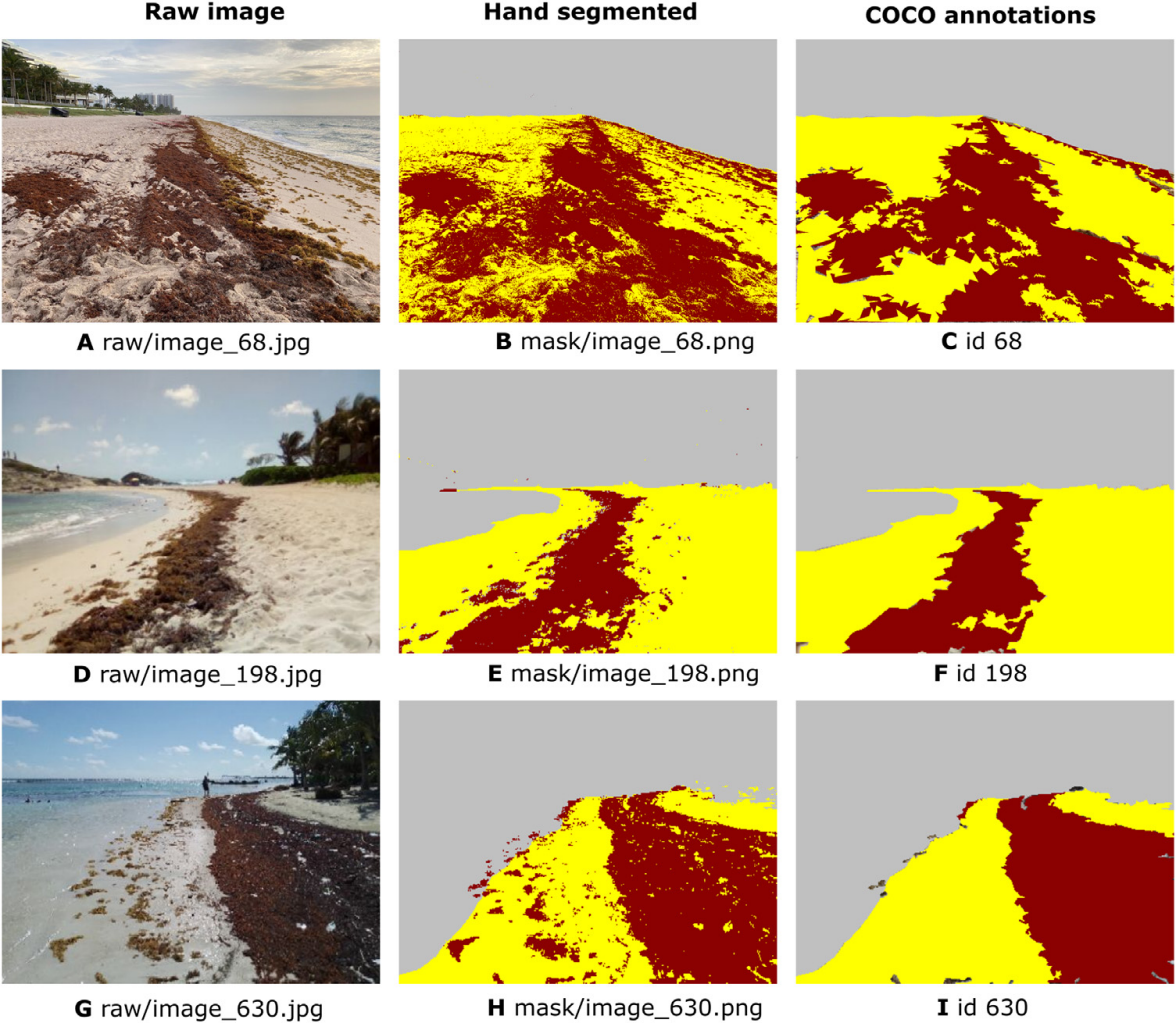
Example of the image dataset showing accumulated *Sargassum* on the beach. Each column represents one of the three different file types that make up the dataset: raw images in .jpg format, manually segmented images in .png format, and COCO images. Image A shows a greater dispersion of *Sargassum* across the beach, resulting in 56 individual polygonal instances for the COCO format (Image C). In contrast, Image D corresponds to four individual polygonal instances (Image F), while Image G corresponds to eight (Image I) [4].

To facilitate immediate use, the segmentation annotations provided in this study follow the structure of the COCO standard [3]. The final JSON file (coco_sargassum_dataset.json) consolidates the metadata, structuring the dictionaries required by modern detection architectures: info (general metadata), licenses, categories (which maps the three environmental classes to their integer identifiers), images (which contains the filename and a unique identifier), and the annotations array. Each entry within annotations details the image identifier, category ID, area, bounding box, and polygon coordinates, which were dynamically processed and simplified using the Douglas-Peucker algorithm to ensure computational feasibility. This multi-format data structure provides a standardized baseline for training, validating, and comparing deep learning architectures focused on semantic and instance segmentation. Fig. 2 depicts examples of the image dataset showing accumulated *Sargassum* on the beach.

The Table 1 describes the exact correspondence between the defined environmental categories (*Sargassum*, Sand, and Other), their RGB pixel values within the semantic segmentation masks, and the integer category identifiers assigned in the COCO annotations.

## 4 Experimental Design, Materials, and Methods

The creation of this dataset involved three stages: The first comprised the manual selection of 1000 images showing *Sargassum* on the beach from the image collection of Collective View, a project that used a crowdsourcing approach to collect photographs of *Sargassum* accumulated on Caribbean beaches [1]. The selected images were those with a clear context, where it is possible to observe the characteristics of the beach containing *Sargassum*.

The second stage involved manually segmenting the selected images to highlight three categories: *Sargassum*, sand, and other elements in the image [2]. The time spent creating the dataset was approximately six months, and the work was carried out meticulously by a person with experience in digital image editing, using the GIMP software (https://www.gimp.org/).

Due to the amorphous nature and texture of stranded *Sargassum*, precise geometric boundaries are often ecologically ambiguous. Therefore, a single-expert annotation strategy was deliberately employed to maintain a consistent subjective baseline across the entire dataset, minimizing variance between annotators. Rather than relying on pixel-level agreement among multiple experts (which is more suitable for discrete objects), the quality and accuracy of these masks are grounded in the wide variance of the texture. The robustness of this single-annotator dataset has been empirically validated in previous applications [2], where generative segmentation models successfully learned texture patterns and generated robust segmentations that effectively mitigated human biases at object boundaries.

### 4.1 Conversion to the COCO format and reduction of geometric complexity

The third stage is the main contribution of this study and consisted of the following: a Python script based on OpenCV was implemented to convert segmentation masks into polygonal annotations compatible with models such as Mask R-CNN, addressing the high fragmentation of *Sargassum* that would otherwise generate an excessive number of micro-polygons. To balance spatial fidelity and computational efficiency, a geometric simplification protocol was applied prior to the generation of JSON files. This included color thresholding in RGB format to obtain binary masks per class (e.g., [85, 85, 85] for *Sargassum*), followed by morphological closing with a 2 × 2 kernel to reduce fragmentation and merge contiguous regions. Outer contours were then extracted, eliminating those with areas of fewer than 100 pixels to reduce noise. Valid contours were simplified using the Douglas-Peucker algorithm (cv2.approxPolyDP), with a dynamic epsilon of 0.1% of the perimeter, which allowed for the reduction of vertices while preserving the overall shape. Finally, the simplified geometries were structured in JSON format according to the COCO standard [3].

In the context of high-resolution images taken at ground level with smartphones, an area of 100 pixels represents a negligible physical footprint (fractions of a square centimeter) that lacks ecological or logistical relevance for early warning systems or coastal cleanup efforts. The removal of these microscopic artifacts was strictly necessary to avoid geometric hyper-fragmentation and ensure the computational viability of the resulting JSON annotations, without sacrificing true and significant instances of *Sargassum*.

### 4.2 Distribution of instances and spatial complexity

To explore the complexity of the dataset and the highly fragmented nature of the coastal environment, instance-level statistics were extracted from the COCO dataset (Table 2). As expected in real-world coastal settings, there is a clear difference in the structural distribution of each class across the images.

**Table 2:** Descriptive statistics for COCO instance-level annotations per image across the entire dataset. The metrics quantify structural complexity and spatial fragmentation for each categorized environmental class (*Sargassum*, Sand, and Other). The distribution highlights the extreme fragmentation of *Sargassum* (which reaches up to 522 polygons in a single patch), compared to the contiguous regions of the background classes.

	*Sargassum*	*Sand*	*Other*
***Mean***	*12.30*	*8.41*	*2.95*
***Std***	*25.15*	*11.91*	*9.36*
**Min**	*1*	*0*	*1*
**Max**	*522*	*127*	*250*
***25%***	*3*	*2*	*1*
***50%***	*7*	*5*	*1*
***75%***	*14*	*10*	*2.25*

**Fig. 3:**
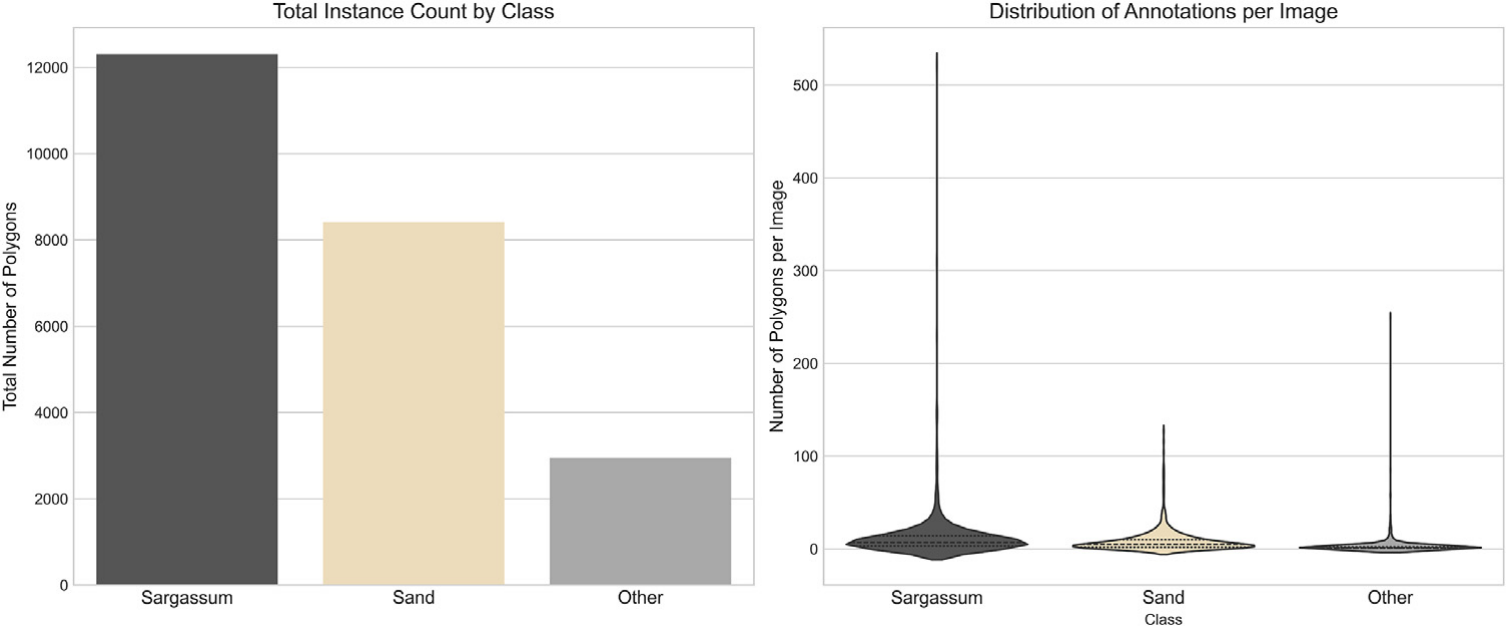
Instance-level statistics and spatial complexity of the COCO-annotated dataset. (Left) Bar chart illustrating the total number of polygonal annotations across the entire dataset (1000 images), demonstrating the natural imbalance between classes, with *Sargassum* accounting for 12,308 individual instances. (Right) Violin plot showing the density distribution of polygons per image for each class. The extended upper tail in the *Sargassum* distribution reflects highly fragmented scenarios, with extreme outliers containing up to 522 discrete polygons within a single image patch.

*Sargassum* exhibits the highest degree of fragmentation, with an average of 12.30 discrete polygons per image patch. Although its median is 7 polygons, the dataset captures extreme cases of *Sargassum* biomass fragmentation, with up to 522 individual polygons within a single image (standard deviation = 25.15). In contrast, the Sand class shows moderate fragmentation (mean = 8.42 polygons) and is the only class that may be completely absent from an image at the instance level (minimum = 0). Finally, the Other class (representing mainly water and vegetation) exhibits the lowest structural complexity, typically consisting of large contiguous regions averaging only 2.95 polygons per image. The heavy-tailed distribution in the *Sargassum* class mathematically highlights the complex variance of the dataset. Furthermore, it provides empirical justification for the geometric simplification protocol (Douglas-Peucker algorithm) applied during the conversion to the COCO phase, which was strictly necessary to ensure computational feasibility and avoid memory bottlenecks in instance segmentation networks (Fig. 3).

The violin plot distribution reveals significant spatial variability. While the median number of *Sargassum* polygons per image is 7, in extreme cases of severe biomass fragmentation, up to 522 individual polygons are observed within a single image patch. This heavy-tailed distribution highlights the challenging intraclass variance of the dataset, underscoring the necessity of the geometric simplification protocol applied during the COCO conversion phase to ensure the computational feasibility of instance segmentation networks.

### 4.3 Pixel-level distribution and semantic imbalance

To better characterize the semantic complexity of coastal landscapes, a pixel-level distribution analysis was performed directly on the segmentation masks. As detailed in Table 3, the dataset exhibits significant intraclass variance and dynamic spatial behavior. The Other class (representing water and background vegetation) generally dominates the visual footprint, exhibiting the highest mean value (382,548 pixels).

**Table 3:** Descriptive statistics of the pixel-level distribution per image across the entire dataset. The metrics quantify the marked spatial variability of the coastal environment, highlighting the situational absence of sand (min. = 0), the predominance of the “Other” class on the seabed, and the extreme variability of *Sargassum* coverage (ranging from scattered patches to massive accumulations covering >12.8 million pixels).

	*Sargassum*	Sand	Other
**Mean**	251,627	137,128	382,548
**Std**	793,788	378,487	1132,990
**Min**	1145	0	964
**Max**	12,888,023	6169,414	15,324,324
**25%**	40,984	28,027	80,361
**50%**	76,320	62,560	112,439
**75%**	168,386	136,171	222,442

**Fig. 4:**
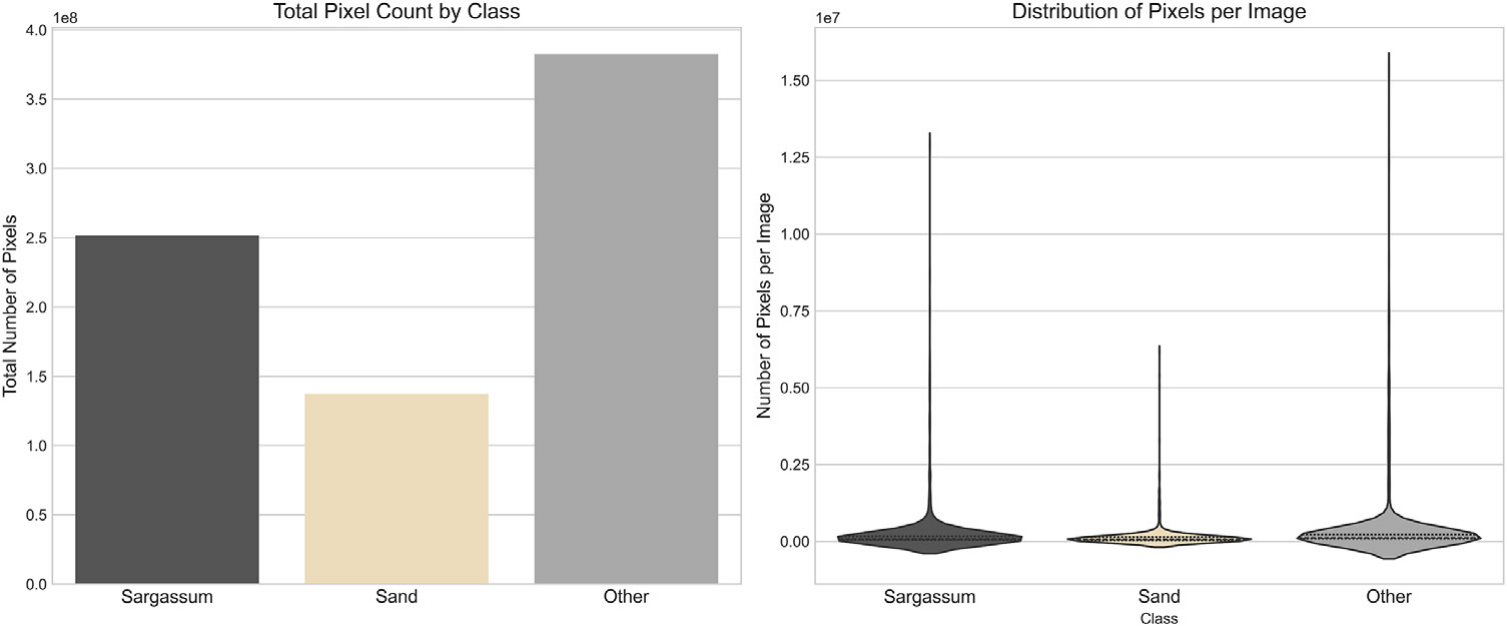
Pixel distribution by class in the dataset of 1000 images. The scatter plot shows high variance. While sand generally has a lower density and clusters near zero for certain images, both the *Sargassum* and “Other” classes exhibit extreme outliers at the upper tail, visually reflecting the highly dynamic and unpredictable nature of biomass accumulations washed ashore.

The Sand class has the smallest average spatial footprint (mean = 137,128 pixels) and is the only class absent in certain images (minimum = 0 pixels). In contrast, *Sargassum* exhibits a highly variable spatial presence. Although its median coverage is 76,320 pixels, the presence of massive coastal accumulations raises the mean to 251,627 pixels, with extreme outliers reaching 12.88 million pixels in a single image patch (standard deviation = 793,788). The correlation of these pixel-level metrics with instance-level statistics (Table 1) reveals the morphological complexity of the dataset. Although *Sargassum* ranks second in average area per pixel, it exhibits the highest degree of fragmentation (with an average of 12.3 polygons per image). This combination, ranging from scattered and highly fragmented micro-patches (min. = 1145 pixels) to massive, continuous accumulations of biomass, serves to create a complex environment (Fig. 4).

## Limitations

Since the dataset is crowdsourced, the images that comprise the dataset were originally captured by volunteers using their smartphone cameras. As a result, the lighting conditions, framing, and focus of the photographs vary. Consequently, the images have different characteristics, and the original files vary in size.

## Ethics Statement

Not applicable.

## CRediT authorship contribution statement

**Javier Arellano-Verdejo:** Conceptualization, Methodology, Software, Data curation, Writing – original draft, Supervision. **Hugo E. Lazcano-Hernandez:** Investigation, Writing – original draft, Writing – review & editing, Visualization. **Arturo Trejo-Morales:** Data curation, Visualization. **Martin Santos Romero:** Data curation.

## Data Availability

FigshareA collection of 1,000 images of beaches with sargassum accumulation, hand-segmented and COCO annotations (Original data).

## Appendix Supplementary materials

Image, application 1

## References

[1] Arellano-Verdejo J., Lazcano-Hernández H.E. (2021). Collective view: mapping *Sargassum* distribution along beaches. PeerJ Comput. Sci..

[2] Arellano-Verdejo J., Santos-Romero M., Lazcano-Hernandez H.E. (2022). Use of semantic segmentation for mapping *Sargassum* on beaches. PeerJ.

[3] Lin T.Y., Maire M., Belongie S., Hays J., Perona P., Ramanan D., Zitnick C.L. (2014). Proceedings of the European Conference on Computer Vision.

[4] Arellano-Verdejo J., Lazcano-Hernandez H., Trejo-Morales A., Santos Romero M.A. (2026).

